# The impact of individual creativity, psychological capital, and leadership autonomy support on hospital employees’ innovative behaviour

**DOI:** 10.1186/s12913-020-05954-4

**Published:** 2020-11-27

**Authors:** Terje Slåtten, Barbara Rebecca Mutonyi, Gudbrand Lien

**Affiliations:** Inland Norway University of Applied Sciences, Campus Lillehammer, 2604 Lillehammer, Norway

**Keywords:** Innovative behaviour, Creativity, Psychological capital, Leadership autonomy support, Hospital, Employees

## Abstract

**Background:**

There is growing interest in and focus on healthcare services research to identify factors associated with innovation in healthcare organizations. However, previous innovation research has concentrated primarily on the organizational level. In contrast, this study focuses on innovation by individual employees. The specific aim is to examine factors with potential impact on individual employee innovation in hospital organizations. Thus, the study significantly deepens and broadens previous research on innovation in the domain of health services.

**Methods:**

A conceptual model was developed and tested on a sample of hospital employees (*n* = 1008). Partial least-squares structural equation modelling (PLS-SEM) was used to analyse the data with SmartPLS 3 software in two steps involving a measurement model and a structural model. Mediation analysis was used to test the proposed indirect effects.

**Results:**

Hospital employees’ individual innovative behaviour is directly and positively associated with individual creativity (β = 0.440), psychological capital (β = 0.34) and leadership autonomy support (β = 0.07). The relationships between leadership autonomy support, psychological capital and individual innovative behaviour are all mediated by employees’ creativity. Psychological capital mediates the relationship between leadership autonomy support and individual innovative behaviour. Overall, the proposed model explains 50% of the variance in hospital employees’ innovative behaviour.

**Conclusions:**

This study reveals a complex pattern of links between innovative behaviour and leadership autonomy support, employees’ creativity and employees’ psychological capital. However, the findings indicate that leadership autonomy support has an influential and multifaceted impact on hospital employees’ innovative behaviour.

**Supplementary Information:**

The online version contains supplementary material available at 10.1186/s12913-020-05954-4.

## Background

Innovation is a desirable objective for successful modern companies. Because innovation is relatively difficult to achieve but considered to be of high value, in many ways it can be said to represent a modern version of the Greek word ‘Eureka’ (which means, ‘I have found it’). Most companies and organizations realize the need to be proactive in their approach to ‘finding it’ or being innovative. Healthcare organizations, whether public or private, are no exception in their desire for innovation. To attain their goals such as organizational efficiency or effective responses to healthcare needs, these organizations consider innovations to play a pivotal role [1]. A current example of this, illustrating the need for innovation, is the Coronavirus disease 2019 (COVID-19) pandemic. Facing this extreme health crisis, health organizations around the world are forced to be innovative for at least two reasons. First, and most obviously, there is an urgent need for a vaccine that hinders or stops the spread of COVID-19. Second, pending a vaccine, health organizations are searching for innovative effective and safe solutions to the ongoing health threat. This latter point is well illustrated in Norway. The Norwegian Institute of Public Health recently introduced an innovative electronic app, named *Smittestopp*, to fight the pandemic. According to the institute, the Smittestopp app ‘will help the health authorities to limit the transmission of coronavirus. Anonymized data about movement patterns in society from the app are used to develop effective infection control measures’ (for more about this app, see [2]). This example demonstrates the need for innovation in healthcare organizations.

Like most organizations, health organizations face constant change and unpredictable challenges [3]. Specifically, healthcare organizations are under continuous pressure to find novel ways to reduce costs and increase the effectiveness of their healthcare services. Because there are various alternative health services to choose from, patients have become more demanding in their expectations for health service quality [4]. These aspects highlight the importance of seeking incremental or radical innovations in almost every area of healthcare. Therefore, it is an urgent need for healthcare organizations to identify and cultivate factors that have a positive impact on innovative behaviour. As Länsisalmi et al. noted, ‘innovation has become a critical capability of all healthcare organizations’ [4].

Although there is a growing body of literature on innovative behaviour in general, very few studies seem to have been undertaken in healthcare organizations. Moreover, in a review on healthcare innovation, Länsisalmi et al. [4] found a large proportion of previous studies (45%) limited their focus to the organizational level of innovations. In their review, the authors found that ‘only 13% of the studies focused on individual level innovations’ [4]. The very few previous studies undertaken have focused on employees’ innovative behaviour in relation to aspects such as employee empowerment and job productivity [5], structural and psychological empowerment [6], motivation and perceived stress [7]. This limited research on individual innovation behavior in healthcare research is surprising because it is reasonable to assume that (individual) employees in organizations are primary and fundamental drivers of the implementation of new ideas, and they are the first to practise innovative behaviour in their work. Xerii and Brunetto, referring to the lack of research on innovative behaviours in healthcare organizations, noted, ‘it is clear that hospitals stand to gain from innovative employees’ [8]. In a similar vein, Kim and Park noted ‘innovative behavior among members of an organization is important … because these individuals are the primary agents to develop and execute innovative ideas’ [9]. Although the literature strongly emphasizes the role of innovation, there is a lack of research on individual innovation in healthcare [4]. Consequently, more research is needed on the potential factors associated with innovative behaviour from an employee perspective in health services research. It is important to point out however, despite creativity being used identically with innovation, in this study, the concept of creativity is separated from that of innovative behavior.

For the reasons above, this paper has three aims. First, an overall aim and contribution is to study innovative behaviour from an employee perspective using healthcare organizations as an empirical setting. Second, according to the literature ‘innovative behaviour [is] influenced by personal characteristics’ [9]. This study addresses two personal characteristics: (i) employee creativity and (ii) psychological capital (PsyCap). According to Yu ‘only a few studies have attempted to determine the impact of PsyCap on employee creativity in the workplace context’ [10]. Third, ‘innovative behaviour is also influenced by … organizational characteristics’ [9]. This study limits its focus to one aspect of leadership. Specifically, it examines whether and how leadership autonomy support is associated with employee PsyCap, creativity and innovative behaviour. By focusing on these three constructs, the study contributes to a relatively neglected domain of health services research.

The paper is structured as follows. First, the conceptual model is briefly described. Second, the content and links between the concepts are discussed. Third, the methods, statistical analysis and results of the empirical hypothesis tests are presented. The paper concludes with a discussion of findings and recommendations for further research. The final part also includes an overall conclusion from this study.

## Conceptual model of the study

**Fig. 1:**
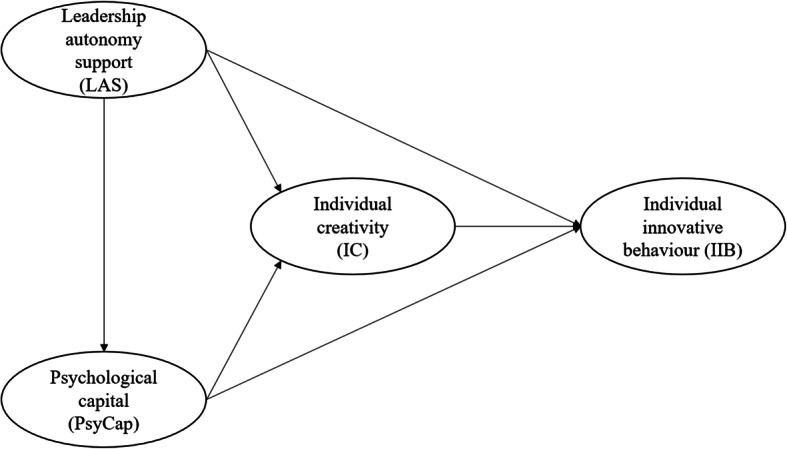
Conceptual model to analyse impacts on hospital employees’ individual innovative behaviour

Figure 1 illustrates the conceptual model. As noted in the introduction, the overall aim of this study is to contribute to research on employees’ individual innovative behaviour (IIB) in healthcare setting.

Figure 1 indicates two distinct types of factors that have an impact on IIB: (i) *personal characteristics* and (ii) *organizational characteristics*. Two *personal characteristics* are represented in Fig. 1: (i) individual creativity (IC) and (ii) PsyCap. PsyCap is assumed to be linked directly to both IIB and IC as well as indirectly to IIB through IC. The *organizational characteristics* represented in Fig. 1 of the conceptual model are labelled ‘leadership autonomy support’ (LAS). LAS is expected to have multiple effects. Specifically, it is assumed that LAS has a direct impact on IIB, IC and PsyCap. Moreover, the linkage between LAS and IIB is expected to be mediated through IC and PsyCap. In addition, the link between LAS and IC is expected to be mediated through PsyCap. All the hypotheses leading this study have been summarized below in Table 1. In the following sections, the concepts and linkages between them, as depicted in Fig. 1, are explained in more detail.

**Table 1:** Hypotheses leading this study

Hypothesis	Hypothesized relationships
H1	IC is positively related to IIB.
H2a	PsyCap is positively related to employees’ IIB.
H2b	PsyCap is positively related to employees’ IC.
H2c	The relationship between PsyCap and IIB is mediated by IC.
H3a	LAS is positively related to IC.
H3b	LAS is positively related to employees’ IIB
H3c	The relationship between LAS and employees’ IIB is mediated by their IC.
H3d	LAS is positively related to employees’ PsyCap.
H3e	The relationship between LAS and IIB is mediated by PsyCap.
H3f	The relationship between LAS and employees’ IC is mediated by PsyCap.

Note: *IC* Individual creativity, *IIB* Individual innovative behaviour, *PsyCap* Psychological capital, *LAS* Leadership autonomy support

### Individual innovative behaviour (IIB)

According to Fuglsang, innovation is ‘a difficult phenomenon to define and study, and there is no consensus about how to define innovation’ [11]. One of the earliest definitions of innovation was that of Schumpeter. Schumpeter refers to innovation as a ‘new combination’ of services, work processes, products and markets [12]. In the literature, an innovation can refer to a ‘new product or service, a new production process, or a new structure or administrative system’ [13]. These diverse definitions of innovation exemplify the potential variety of differences between various types of innovation. Simply stated, innovation can manifest everywhere in an organization. However, this study limits its focus to innovations relevant to individual employees. The innovation type evaluated in this study is IIB in healthcare settings. IIB concerns the implementation of innovations of potential benefit to employee performance. IIB relates to the behaviour of employees and their ability to adopt and use new and useful ideas in their work environment [14]. As such, IIB is doing something new that represents a behavioural change or discontinuity relative to the ordinary organizational pattern of behaviour in the past. Consequently, the domain of IIB is related to everyday employee practices, and such innovations are implicitly ‘a function of learning and knowledge creation, integrated into daily work practices’ [15]. Furthermore, there is no explicit focus on the timing of implementation. Innovation may be implemented either as a one-time change (e.g. for a specific patient or situation) or more permanently (e.g. a new procedure that is extended to all future patients). Innovation in a one-time situation is analogous to what the literature terms an ad hoc *innovation* [16]. Similar to ad hoc innovation, IIB may include some temporary innovations. However, the concept of IIB can include ‘some element that can be repeated in new situations’ [17], to be implemented and generalized more permanently. Consequently, the concept of IIB in this study is open to a wide range of changes relevant to employee performance. Thus, IIB embraces and reflects a ‘ … specific form of change-oriented activity’ [18] that is explicitly manifested in employees’ ‘implementation of new and useful ideas within a work-role’ [18]. Below, some significant factors suggested to have an impact on IIB are addressed.

### Individual creativity (IC)

As shown in the conceptual model in Fig. 1, IC is one of two personal characteristics suggested to have an impact on IIB. IC as a personal characteristic reflects the idea that creativity is heterogeneous and distributed across individuals in organizations. Creativity is flexible and dynamic; it varies from one employee to another. Therefore, IC is an individual resource or capability to be creative. Based on this, and specifically for this study, IC is defined as the individual employee’s ‘production of novel, useful ideas or problem solutions. IC refers to both the process of idea generation or problem solving and the actual idea or solution’ [19]. Creativity is sometimes used synonymously with innovation. However, in this study, we separate the concept of IC from that of IIB. Shalley et al. support this distinction, stating: ‘it important to distinguish creativity from innovation. Creativity refers to the development of novel, potentially useful ideas. Although employees might share these ideas with others, only when the ideas are successfully implemented at the organization or unit level would they be considered innovation’ [20]. As the above definition suggests, IC refers to the production and development of potentially useful and novel ideas. Consequently, IC describes processes and individual cognitive thoughts (referring to creative thinking) and potential associated activities such as (1) defining the problem to be solved, (2) collecting information, (3) generating ideas and (4) evaluating ideas [21]. In contrast to IC, the concept of IIB relates to behaviour, specifically referring to the behavioural implementation of creative ideas. Consequently, there is a natural distinction between IC and IIB, although the two concepts are closely linked or interdependent.

Creativity is most often described as a necessary ‘input’ to innovation. Slåtten and Mehmetoglu, emphasizing the importance of creativity, characterized it as a ‘primary source’ [22] of innovative behaviour. Gilmartin illustrates the criticality of creativity by describing it as ‘the fuel of innovation’ [23]. The ‘foundation of innovation ideas is creativity’ [24]. Previous research has found a positive link between creativity and innovation at the individual level [22]. In line with previous research, this study sought a positive association between IC and IIB. This leads to the following hypothesis:

- **Hypothesis 1:** *IC is positively related to IIB.*

### Psychological capital (PsyCap)

PsyCap in Fig. 1 is the second personal characteristic that may influence IIB. The PsyCap construct is drawn from positive psychology, and concerns ‘who you are’ as a person [25]. More precisely, PsyCap focuses on the positive aspects and strengths of individuals and labels them collectively as positive psychological resources [26] for the innovative process. Luthans et al. described PsyCap as a higher order construct, which encompasses four first-order positive psychological resources: (i) hope, (ii) self-efficacy, (iii) resilience and (iv) optimism [26]. All four resources included in PsyCap are state-like resources [25]. The hope dimension in PsyCap is a motivational state that describes the extent to which one can progress when facing obstacles. Self-efficacy is individual confidence in one’s ability to perform tasks successfully. Resilience refers to the capability to manage setbacks, pursue objectives and achieve good results. Optimism is a person’s positive assessment of the future [27]. This defines PsyCap consistent with previous research as an individual’s positive psychological state of development characterized by (1) having confidence (self-efficacy) to take on challenging tasks and put in the necessary effort to succeed at them; (2) having a positive feeling (optimism) about future success; (3) persevering towards goals, and when necessary redirecting paths to goals (hope) to succeed; and (4) when beset by problems and adversity, bouncing back, sustaining or increasing one’s efforts (resilience) to attain success [27].

Previous research has associated individual PsyCap with work related performance, including IIB. For example, Slåtten et al. found that PsyCap among service sales employees was positively associated with innovative behaviour [28]. In another study, Abbas and Usman found a positive link between PsyCap and supervisor-rated innovative performance among employees employed in a range of fields [29]. Research has also found that the individual components and resources of PsyCap are linked to innovative behaviour. For example, research has linked the single PsyCap component of self-efficacy to innovative activities [30] and creative performance [31]. Although this study focuses on the collective impact of all (four) resources of PsyCap and does not examine the impact of single components, it supports the assumption of a link between PsyCap and IIB. In line with most previous research, it is expected that PsyCap in such settings will ‘provide a necessary repository of psychological resources that help effectively innovative work-related ideas’ [29]. Based on this, the following hypothesis is proposed:

- **Hypothesis 2a:** *PsyCap is positively related to employees’ IIB.*

Although it has been suggested that PsyCap has a direct impact on IIB, it is also reasonable to assume that PsyCap has an additional direct impact on IC. Previous research has revealed that IC is linked to personal factors [32]. In this study, PsyCap reflects these individual factors. Specifically, it is expected that PsyCap is not limited to its positive impact on an individual employee’s adoption of an innovation (referring to IIB) but also of triggering creativity (referring to IC). It is important to remember that IC in the previous discussion was described in terms such as ‘primary source’ ([22] and ‘foundation of innovation’ [24]. Simply and directly stated, without creative thoughts, no innovative behaviour will occur. Gilmartin supports this assumption, stating, ‘creativity is the basic building block of invention and thus innovation’ [23].

Each of the four resources of PsyCap is a potential enabler and helps to trigger IC. Creative thinking is not a quick fix but often involves extensive and intensive cognitive and mental work. It is reasonable to assume that the mental work of IC entails some form of learning process of at least four steps. First, a person must be aware of a problem or challenge that needs to be solved. Second, a person must be interested and motivated to explore the nature of the problem (‘What is the real problem to be solved here?’). Third, potential solutions are identified. In this part, there may be several and sometimes even competing solutions, each with its specific obstacles. Fourth, among the list of alternative solutions, one must finally evaluate and identify the most appropriate and practical solution. Based on this four-step IC process, it is easy to imagine that IC is a relatively demanding mental/cognitive process that can be frustrating, time-consuming and stressful. However, a person’s PsyCap resources can boost IC. PsyCap is a core resource to achieve IC because it represents ‘one’s positive appraisal of circumstances and probability for success based on motivated effort and perseverance’ [26].

Previous research has revealed that the four resources or ‘ingredients’ of PsyCap, both individually and collectively, are associated with IC [33, 34]. For example, previous research has linked the hope resource of PsyCap to a person’s will to perform creative exploration [35]. Luthans et al. explicitly stated that hopeful employees ‘tend to be creative’ [35]. Similarly, in regard to optimism Rego et al. found that optimistic people tend to be more creative than their less optimistic counterparts [36]. Research on the other two resources of PsyCap, self-efficacy and resilience, has also found them to be positively linked to the aspect of creativity (see e.g. [30, 31, 37]). Consequently, the four resources of PsyCap are all potentially associated with IC. Scarce research has examined the impact of PsyCap on employees’ IC in a healthcare setting, making this study a unique contribution to health services research. Based on previous research, it is expected that the ‘combined motivational effects of the four dimensions’ [33] of PsyCap will be positively associated with employees’ IC. The assumption about this relationship can be summarized in the following hypothesis:

- **Hypothesis 2b:** *PsyCap is positively related to employees’ IC.*

The two aforementioned hypotheses propose that PsyCap has a direct impact on employees’ IIB and IC. However, as shown in Fig. 1 and summarized in Table 1, it is also expected that the relationship between PsyCap and IIB is mediated by IC. This assumption represents a third alternative way in which PsyCap may be linked to IIB. The main argument for this third route of impact is in the core role IC seems to have in IIB. As emphasized above, IC in the literature is described as a ‘primary source’ [22] and the ‘foundation of innovation’ [24]. This implies that from an individual employee perspective, IC is a necessary precondition for IIB. Based on this core role of IC, an increase in employee IC because of a positive shift or change in their PsyCap (as suggested in hypothesis 2b) may encourage employees to experiment with and apply creative ideas if they see a benefit to their work. Consequently, IC is expected to mediate between PsyCap and IIB. This leads to the following hypothesis:

**Hypothesis 2c:** *The relationship between PsyCap and IIB is mediated by IC.*

### Leadership autonomy support (LAS)

In the conceptual model in Fig. 1, LAS represents *organizational characteristics.* In general, leadership is an essential organizational variable because it influences employees’ psychological attributes (e.g. PsyCap) and their creative performance [38] in constructs such as IC and IIB. LAS may affect motivation in work contexts [39]. This motivation is interesting for two reasons. First, as mentioned above, IC and IIB are relatively stressful and demans action. Second, IC and IIB can both be described as ‘extra-role behaviour’ because they are normally not a direct obligation, nor are they explicitly stated in formal contracts or job descriptions. Therefore, creative performance in terms of IC and IIB can be described as voluntary hard work that employees want to do but do not have to. Consequently, employees need a certain level of interest, or more precisely, motivation to be creative and innovative. This latter aspect of employee motivation is interesting and especially relevant to the concept of LAS. The ideas in this concept originally come from self-determination theory (SDT) [40]. SDT focuses on factors that facilitate motivation in humans. In SDT, the inner or self-determined driven type of motivation is emphasized as the ideal type. In SDT, it is labelled ‘autonomous motivation’, which describes a person who ‘behaves with a full sense of volition and choice’ [41]. In the literature, autonomous motivation is described as the ‘highest quality of regulation’ [41], and is closely linked to LAS [41, 42]. Hence, LAS is of special interest to the overall aim of this study.

In this study, LAS refers to employees’ perceptions of the quality of their interpersonal relationship with their leader. The domain and focus of LAS is the interpersonal work context and whether employees perceive their leader as one who stimulates, motivates and encourages them to work autonomously. Leaders that are autonomy-supportive provide ‘a meaningful rationale for doing the task, emphasise choice … and acknowledge employees’ feelings and perspective’ [41]. The ‘goodness’ and ‘well-being’ of autonomy-supportive leaders become very clear if it is contrasted with the opposite—non-autonomy-supportive leaders. In an organization with non-autonomy-supportive leaders, employees have minimal or zero freedom, are controlled at every step of the way, and their leaders give orders and provide detailed recipes of how the work should be done. Not surprisingly, employees most often feel that non-autonomy-supportive leaders decrease their inner motivation while autonomy-supportive leaders increase it. Therefore, because autonomy at work and autonomy-supportive leaders are closely associated with employees’ inner motivation, they are most often appreciated and sought by employees. Individuals who seek autonomy at work ‘are often searching for inner motivational environments and situations that provide them the opportunity of self-determination, initiative and choice’ [43].

There are several interconnected reasons why LAS should have a direct impact on both employees’ IC and employees’ IIB. First, LAS potentially ‘fuels’ employees with an inner motivation that increases their interest and leads them to focus on their work performance. Previous research supports the view that autonomy support is linked to employee motivation in work contexts. Second, because LAS is associated with positive motivation, it is reasonable to assume that employees also become more engaged and dedicated, which increases their IC and their IIB. Consequently, by this reasoning, employees’ perceptions of LAS function in tandem with their motivation by promoting IC and IIB. The importance of motivation for creativity and innovation is supported in the componential theory of creativity. By this theory, the motivation of an individual is suggested to be a primary mechanism that affects the creativity of an individual [44]. Furthermore, the creativity of an individual is noted as an predecessor for IIB at work, as the generation of ideas (creativity) is a necessary step towards the implementation (innovation) of ideas [45, 46]. As noted by Hocine and Zhang, ‘people are most creative when they feel motivated’ [47]. Previous research suggests that autonomy-supportive leaders have an impact on employee performance [44, 47]. Frese and Zapf, for example, found that the more leaders encouraged and supported employees in organizations, the more it promoted new ideas, creativity and the implementation of those ideas [48]. In an empirical study by Slåtten including 345 hospitality employees, the author found that their perceived autonomy influenced both their creative self-efficacy and innovative behaviour [30]. In this paper, the authors suggest that autonomy is a ‘ … “key factor” and is critical for developing a person’s creative self-efficacy’ [30]. Previous research has also revealed that when employees experience the opposite of autonomy at work—controlling behaviour from their leader—this has a detrimental impact on creativity and innovation [49]. Consequently, based on previous research, there are several good reasons to assume that when employees perceive LAS in a positive way it will have a positive impact on both IC and IIB. This reasoning leads to the following hypotheses:

- **Hypothesis 3a:** *LAS is positively related to IC.*
- **Hypothesis 3b:** *LAS is positively related to IIB.*

Shalley et al. state that ‘the presence of … creative ideas increases the likelihood that other employees will apply the ideas in their own work’ [20]. This statement—like the present study—stresses the importance of IC in achieving IIB. Consequently, creative thinking (or IC) is a precursor for creative acting (or IIB). On the other hand, as previously mentioned, there are studies revealing that autonomy is positively associated with innovative behaviour [45] and creative work involvement [46]. However, in these studies, the impact of autonomy is limited because they do not include both IC and IIB in the same study. Therefore, considering the core role of IC, the true pattern of linkages in the impact of autonomy on IC and IIB has not been fully investigated. In contrast, this study separates IC (as a cognitive concept) from IIB (as a behavioural concept), thus providing a more comprehensive test for mechanisms operating between LAS, IC and IIB. Previous research has yet to explore the linkages between these three concepts. Being creative is demanding for employees and it entails abilities such as ‘deep processing of information, and information integration’ [50]. Thus, being creative is a complex task. Such ‘complex tasks or quality-type tasks tend to require a higher degree of engagement and autonomy’ [50]. LAS is, therefore, a leadership tool that may increase employees’ IC. Based on this, when employees perceive the LAS to be good it should encourage them and stimulate their IC. However, LAS is not limited to raising employees’ creative thinking skills. It is also reasonable to assume that LAS, in the next round can fuel employees with the necessary authority and freedom to transform their creative thoughts (IC) into real action and behaviour (IIB). This is because implementing creative thoughts may benefit work performance. This reasoning assumes that IC acts as the common denominator between LAS and IIB. Specifically, IC is expected to mediate the relationship between LAS and IIB. This leads to the following hypothesis on the pattern of linkages:

- **Hypothesis 3c:** *The relationship between LAS and employees’ IIB is mediated by their IC.*

Because of leaders’ and managers’ formal roles in organizations, they significantly influence their subordinates [51]. Slåtten et al. describe this influence as ‘among the most dominant factors’ [52]. Leadership is a significant or ‘impactful’ part of an organizational work environment and ‘resource theorists view the work environment as a key management resource that interacts with other resources’ [53] such as the resources that comprise PsyCap. As discussed in relation to hypotheses 3a and 3b, leadership is expected to affect employees’ IC and IIB. Below, it is suggested that this relationship also works through the impact of LAS (an *organizational characteristic)* on PsyCap (a *personal characteristic*) as shown in Fig. 1 and summarized in Table 1. Consequently, this represents an alternative and complementary route in the pattern of linkages associated with IC and IIB.

The literature defines the concept of PsyCap as ‘an individual’s positive psychological state’ [27]. The definition of it as a ‘psychological state’ implies that PsyCap is not static or fixed but flexible and dynamic. Consequently, the individual resources that comprise PsyCap change according to certain factors. Luthans et al. support this idea by stating that PsyCap is ‘open to development and can be managed for effective work performance’ [25]. By this line of reasoning, it is expected that LAS can positively ‘develop’ or ‘manage’ employees’ PsyCap. Current research has yet to examine this specific relationship in a healthcare setting. Although very little research has been undertaken, previous research indicates a relationship between LAS and PsyCap. First, when employees perceive the LAS in their organization to be positive it reflects a perception of an autonomous work environment. As discussed above, an autonomous work environment (of which LAS is a part) is positively associated with PsyCap. For example, in a study by Choi including 331 employees in a Korean automotive parts manufacturing company, the author found a significant and strong link between autonomous work environments and employees’ PsyCap (β = 0.586) [53]. Interestingly, in this article the author describes an autonomous work environment as partly a place that ‘gives employees choices and encourages employees to take personal initiative’ [53]. Moreover, to capture employees’ perceptions of autonomy the author’s questionnaire used items that assessed ‘a subordinate’s perceptions of the degree of autonomy supportiveness provided by their supervisors’ [53]. This way of describing and capturing autonomous work environments is to a large extent similar to how the concept of LAS is used in this study. Stated in another way: Choi provided support for this study’s expectation of a positive association between LAS and PsyCap [53]. Second, although the impact of LAS has not been specifically considered, previous research found that positive leadership (e.g. authentic leadership) and supportive organizational climate are positively associated with PsyCap [28, 54]. Consequently, based on the highly relevant research of Choi [53], it is expected in this study that LAS, as a positive environmental resource in organizations, has a positive impact on employees’ PsyCap. Therefore, the following hypothesis is proposed:

- **Hypothesis 3d:** *LAS is positively related to employees’ PsyCap.*

Innovative behaviour is influenced by both ‘personal and external determinants’ [55]. As argued throughout the discussion of the previous hypothesis, both PsyCap and IC—two personal determinants (or *personal characteristics)*—are assumed to be positively associated with IIB. Moreover, in the discussion of hypothesis 3d, it was argued that LAS, as an external determinant (or *organizational characteristic)* develops and increases the ‘reservoir’ of employee PsyCap resources. Based on this reasoning and assumption, it is reasonable to assume that PsyCap plays a mediating role in the relationship between LAS and IIB. Specifically, when employees’ reservoirs of PsyCap increase because of a positive development stemming from LAS, this should increase their IIB. The authors are not aware of any previous study that has specifically tested the interplay between these variables in healthcare settings. However, a previous study has found that PsyCap mediates the relationship between positive leadership and innovative behaviour. For example, in a study of sales-people, it was found that employees’ PsyCap mediated the relationship between positive perceptions of the authentic leadership style and innovative behaviour [28]. Furthermore, Choi found that PsyCap fully mediates the relationship between an autonomous work environment (of which LAS is a part) and employees’ self-directed behaviour (a concept that is strongly related to the concept of IIB in this study) [53]. Thus, given its prominent role reported in the literature, PsyCap is expected to mediate the relationship between LAS and IIB. This prompts the following hypothesis:

- **Hypothesis 3e:** *The relationship between LAS and IIB is mediated by PsyCap.*

A similar mediating pattern of linkages with PsyCap is predicted between LAS and IC. In this study, IC is defined as a cognitive concept. As noted several times in this paper, creativity is fundamental as the first step to innovation [20, 22, 23]. The logic of this is simply stated: If an individual has no creative thoughts (IC) no innovation will occur (IIB). However, as stated in hypothesis 2b, PsyCap can fuel IC. Similarly, as argued in hypothesis 3d, LAS can fuel PsyCap. In combination, these relationships indicate mediation or what can be described as a ‘domino effect’ that starts with perceptions of LAS, works through PsyCap and has an impact on IC. Scarse previous research has examined this assumption in a healthcare setting. However, support for the hypothesized mediating relationship can be found in published studies. Gupta and Singh found in their study that PsyCap fully mediates the relationship between leadership and creativity [56]. Similarly, Zubair and Kamal found that PsyCap mediates the relationship between the authentic leadership style and creativeness [57]. In line with previous research, it is assumed that PsyCap mediates the relationship between LAS and IC. This leads to the following and final hypothesis in this study:

- **Hypothesis 3f:** *The relationship between LAS and employees’ IC is mediated by PsyCap.*

## Methods

The focus of this paper is the IIB of hospital employees. One of the authors initiated contact with the Director of Research at a hospital located inland in Norway to request permission to survey its employees. After acceding to the request, the Director of Research informed the hospital staff unit, the hospital division managers and the hospital department managers about the project. Both the division managers and the department managers undertook to inform the employees in their divisions and departments.

The study was submitted to and approved by the Norwegian Social Science Data Services (NSD), and the Data Protection Officer at the hospital. An informed consent letter was later issued by e-mail to all participants of the study, and was also included on the first page of the questionnaire. All invitations included information about the aim and focus of the study, confidentiality of data, voluntary participation and the estimated time required to complete the questionnaire. Participants were required to give their consent before participating in the survey.

The survey was developed through several workshops, a meeting with experts from academia and the site of the study. This process included several pretests of the questionnaire. Based on the feedback from the pretests, some redundant or ambiguous items were modified or deleted. The final questionnaire was distributed to a sample of 2000 hospital employees across seven staff units and 10 divisions. The selection of the staff units and divisions was made by consultation between the Director of Research, human resource management office and senior hospital management. It was the Director of Research who first disseminated the survey through emails to the hospital division managers and the hospital department managers. Then, in the next round, the managers distributed the questionnaire to the employees in their division. There were two reasons for this. First and foremost, we were able to ensure full anonymity through the platform Nettskjema (www.nettskjema.no) as no e-mail addresses were obtained. Second, because of the complexity of healthcare systems in general, obtaining data can be challenging. As such, healthcare managers (staff unit managers, division managers and department managers) were viewed as great ambassadors who would encourage and motivate employees to participate in the study. A sample size (*n* = 1008) of hospital employees participated, which gave a response rate of 50.4%.

Table 2 provides information on the personal characteristics of the participants. Because this study focused on hospital employees as a whole, further modifications in personal characteristics of the participants were made. First, in LAS, the term ‘leader’ for respondents meant their immediate formal leader. Second, all specialized positions or roles were summarized in their respective categories; for example, specialized nurse was summarized in the Nurse category, and specialized doctor, was summarized in the Doctor category. It is noteworthy that this study made no distinction between roles, but focused on all hospital employees employed at the study hospital, regardless of their rank and work title.

**Table 2:** Personal characteristics of the participants (*N* = 1008)

		%
Sex:	Female	73.0
	Male	27.0
Staff role:	Nurse	33.0
	Doctor	8.7
	Others (e.g. admin. Staff, other health professionals)	58.3
Duration of employment:	Less than 5 years	26.9
	Between 6 and 10 years	18.0
	Between 11 and 20 years	30.3
	More than 20 years	24.8
Part-time or full time:	Part-time	22.5
	Full time	77.5
Age:	Younger than 45 years	37.3
	Between 46 and 55 years	32.2
	Older than 55 years	30.5

### Instruments

This study covered four constructs: LAS, PsyCap, IC and IIB. All items used for the constructs are based on previous research. However, because none of the instruments has specifically been used in a structured healthcare analysis studies before, there was a need to adapt items into the study context from previous interdisciplinary studies. The items used to capture the concept of LAS were adopted from Amundsen [43]. The items used to capture the concept of PsyCap were adopted from Luthans et al. [27]. Those for IC items were adopted from Zhou and George [58]. Finally, the IIB were adopted from Janssen [59] and Scott and Bruce [60]. It is important to note that the LAS construct and items included in this study have only been validated in a non-healthcare setting [43]. The PsyCap, IC and IIB constructs have previously been validated in healthcare settings [61–63], but have not been used in a structured analysis, such as in this study. All items included in this study were therefore adapted to fit the healthcare setting in the Norwegian context. The concise definition of the adopted concepts and their items are summarized in Table 3. A Likert scale from (1) strongly disagree to (7) strongly agree was used for all items. The survey used in this study is a part of a larger survey research project focusing on various aspects of employee-relations in health organizations. As such, claims used in this study are appended accordingly (see appendix 1).

**Table 3:** Constructs (LAS, PsyCap, IC and IIB) and items used in this study

Construct	Definition	Claims label	Claims	Source
LAS	LAS refers to employees’ perceptions of the quality of their interpersonal relationship with their leader.	LAS1	My leader gives me authority over issues within my area.	Amundsen [43]
		LAS2	My leader listens to me.	
		LAS3	My leader encourages me to take initiative.	
		LAS4	My leader is concerned that my work is goal-oriented.	
		LAS5	My leader instils motivation.	
PsyCap	An individual’s positive psychological state of development characterized by self-efficacy, optimism, hope and resilience.	PsyCap1	I feel confident that I can set goals for myself in my work area.	Luthans et al. [27]
		PsyCap2	I am optimistic when it comes to my future at this organization.	
		PsyCap3	When faced with challenges in my job, I can find alternative solutions to them.	
		PsyCap4	I can find alternative ways to achieve my goals.	
IC	The individual employee’s ‘production of novel, useful ideas or problem solutions. It is both the process of idea generation and the actual idea.	IC1	I contribute creative ideas to solve challenges in my job.	Zhou and George [58]
		IC2	I contribute creative ideas to improve the quality of my job.	
IIB	The behaviour of employees and their ability to adopt and use new and useful ideas in their work.	IIB1	I create new ideas to solve problems in my job.	Jansen [59] and Scott & Bruce [60]
		IIB2	I search out new working methods or techniques to complete my work.	
		IIB3	I investigate and find ways to implement my ideas.	
		IIB4	I promote my ideas so others might use them in their work.	
		IIB5	I try out new ideas in my work.	

Note: *LAS* Leadership autonomy support, *PsyCap* Psychological capital, *IC* Individual creativity, *IIB* Individual innovative behaviour

### Data analysis

Partial least-squares structural equation modelling (PLS-SEM) was used to test the conceptual models and hypothesized relationships, using SmartPLS 3 software [64]. As a first step in evaluating the PLS-SEM results, a set of criteria for the reflective measurement model was assessed; the second step involved evaluating the structural model. Next, we estimated and analysed the hypothesised mediating effects. By following the recommended steps of Hair et al. [65, 66], we were able to assess the quality of the measurement model results and the structural model results.

As a robustness check of the PLS-SEM results, we also tested whether the following socio-demographic control variables influenced IIB: age, sex, education level and type of employment (part-time or full time). No significant differences were found for the socio-demographic variables, so the control variables were excluded from further analysis.

## Results

### Measurement model

In evaluating the reflective measurement model, we followed the recommendations of Hair et al. [65] by including the following: convergent validity, internal consistency reliability and discriminant validity. In short, convergent validity is measured by the average variance extracted (AVE), and estimates the average variance shared between the studied constructs and their individual indicators. As reported in Table 4, all loadings were above the recommended criterion of 0.7. In addition, the constructs in this study demonstrated AVE values well above the recommended 0.5. Therefore, we could conclude that the measurement model exhibited a satisfactory degree of convergent validity. Further, the reliability of the construct, internally, includes both the composite reliability and the Cronbach’s alpha. In short, internal consistency reliability is an estimate that show whether the individual claims are all measuring the same construct, creating issues of redundancy. The results of our measurement model, as indicated in Table 4, revealed a good internal consistency in the constructs with values above the recommended 0.7. Lastly, we tested the measurement model for distinctiveness of the studied constructs. As suggested by Hair et al. [65, 67], we used the heterotrait–monotrait (HTMT) to reveal whether the shared variance within the studied constructs, their AVE, exceeded the shared variance between the studied constructs. As shown in Table 4, the 95% confidence interval of the HTMT statistic, did not include values of 1, signifying that discriminant validity was present. Overall, the tests suggested that the proposed reflective measurement model in this study is reliable and valid.

**Table 4:** Results of the measurement model for the LAS, PsyCap IC and IIB constructs

		Convergent validity		Internal consistency reliability		Discriminant validity
Construct	Claims label	Indicator reliability	AVE^a^	Composite reliability	Cronbach’s alpha	HTMT criterion^a^
‘Rule of thumb’		Loading > 0.7	> 0.5	0.7–0.95	0.7–0.95	HTMT interval does not include 1
LAS	LAS1	0.84	0.80	0.95	0.94	Yes
	LAS2	0.92				
	LAS3	0.93				
	LAS4	0.86				
	LAS5	0.91				
PsyCap	PsyCap1	0.81	0.74	0.92	0.88	Yes
	PsyCap2	0.82				
	PsyCap3	0.89				
	PsyCap4	0.90				
IC	IC1	0.96	0.93	0.96	0.92	Yes
	IC2	0.96				
IIB	IIB1	0.85	0.77	0.94	0.93	Yes
	IIB2	0.88				
	IIB3	0.90				
	IIB4	0.88				
	IIB5	0.88				

^a^*AVE* Average variance extracted, *HTMT* Heterotrait–monotrait ratio of correlations

### Structural model

**Fig. 2:**
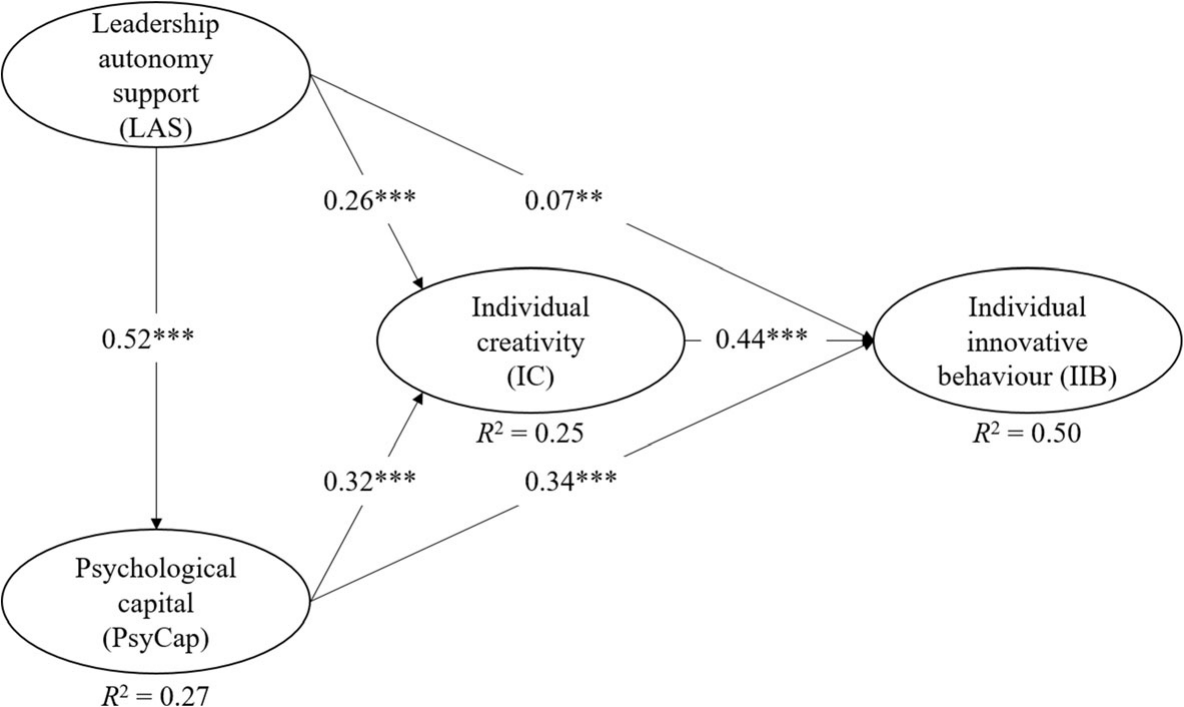
Results of the structural model of the effect of leadership autonomy support, PsyCap and creativity on hospital employees’ IIB. Standardized coefficients (** < 0.05, *** < 0.01)

Seeing as the reflective measurement model was confirmed, we then continued to assess the studied structural model. We first evaluated the studied constructs to determine multicollinearity issues. Following the recommended steps of Hair et al. [67], we examined model collinearity issues by observing the variance inflation factor (VIF), to ensure all VIF values were below 3. The results of the structural model collinearity revealed VIF values below 2, suggesting no multicollinearity issues. As such, it allowed us to examine and test the size and significance of the proposed path coefficients, as shown in Fig. 2. In addition, to measure the structural model prediction, we assessed the in-sample prediction of all endogenous constructs using *R*^2^. Following the suggestions of Hair et al. [65, 67], the *R*^2^ values for IIB (0.50), PsyCap (0.27) and IC (0.25) were moderate. The path coefficients values were standardized and revealed statistically significant values at the 1% significance level (the coefficient between LAS and IIB at the 5% level). The relationship between IC and IIB was positive (b = 0.44), supporting H1. H2a and H2b were also supported because the relationships between PsyCap and IIB and between PsyCap and IC were positive (b = 0.34 and b = 0.32, respectively). Finally, the structural model revealed a positive relationship between LAS and PsyCap (b = 0.52), between LAS and IC (b = 0.26) and between LAS and IIB (b = 0.07), supporting H3a, H3b and H3d.

The test of the mediator effects shows that IC complementarily mediates the relationship between PsyCap and IIB, with an indirect effect of 0.14 (Table 5), supporting H2c. Furthermore, IC intervenes between LAS and IIB (indirect effect of 0.11), supporting H3c, and PsyCap also complementarily mediates the relationship between LAS and IIB (indirect effect of 0.18), supporting H3e. Note the ‘domino effect’ that PsyCap and IC have on the relationship between LAS and IIB, with an overall indirect effect of 0.36. The mediating effect of PsyCap between LAS and IC was 0.17, also indicating a complementary mediator effect, supporting H3f. We used the bootstrapping test of Zhao et al. [68] to test mediation. Briefly, this test uses bootstrapping to assess how a third variable intervenes between two related constructs [65], and whether the direct and indirect effects are statistically significant. As such, the combination of these two tests determines whether there exist direct effects only—without mediation, no-effect non-mediation, complementary mediation, competitive mediation or indirect-only mediation.

**Table 5:** Test of mediation effect of IC and PsyCap

Hypothesis	Effect^a^	Mediator	Indirect effect^a^	Total effect^a^	Mediator effect^b^
H2c	PsyCap → IIB	IC	0.138^***^	0.477^***^	Complementary
H3c	LAS → IIB	IC	0.114^***^	0.436^***^	Complementary
H3e	LAS → IIB	PsyCap	0.176^***^	0.436^***^	Complementary
H3f	LAS → IC	PsyCap	0.165^***^	0.427^***^	Complementary
^c^	LAS → IIB	IC, PsyCap	0.362^***^	0.436^***^	Complementary

^a^** *p* < 0.05, ***p < 0.01 are significance levels

^b^The effect between LAS and IIB is influenced by two mediators, IC (twice) and PsyCap, and we have a triple mediation analysis [65]. The total indirect effect is then the sum of the specific indirect effects

^c^Mediation by bootstrapping method [68]

## Discussion

Innovation is a ‘critical capability of all healthcare organizations’ [4]. This study aims to increase our understanding of the foundations of innovation in healthcare organizations. The contributions can be summarized in three parts. First, in contrast to most previous research at the organizational level of innovation, this study focuses on innovation from an individual employee perspective. As such, it deepens our insight into employees in healthcare organizations that the literature sometimes describes as ‘primary agents’ [9] of innovative ideas. Second, previous health services research has been limited to the behavioural manifestations of innovation or what this study labels IIB. Although IIB is an interesting aspect, this study extends previous research as it increases our knowledge regarding factors that have an impact on employees’ cognitive processes associated with IIB. By including the concept of IC, this study offers insight into the links between the fundamental premises of IC and IIB. Third, this study also examines whether and how IIB is manageable. Specifically, it reveals how organizational factors (or LAS) combine with personal factors (PsyCap and IC) influence IIB. Consequently, in summary, the study unpacks the apparent ‘black box’ by revealing a multifaceted pattern of linkages that make up employees’ IIB.

In line with previous research, IIB in this study was defined as ‘implementation of new and useful ideas within a work role’ [18]. As mentioned above, IIB embraces a variety of behavioural manifestations of ‘newness’ at work. Specifically, ‘newness’ ranges from incremental (minor) innovations on one hand to radical (major) innovation on the other. Similarly, the aspect referred to as ‘within’ a work role in the definition of IIB embraces a great variety of ‘time and places’ where ‘newness’ or innovation take place. Specifically, ‘within’ a work role could include innovation by frontline employees (e.g. finding a new way to manage patients) as well as ‘within’ backstage work (e.g. a new administrative routine or internal work process). Thus, the definition of IIB in this study touches on one of the earliest definitions of innovation, provided by Schumpeter [12], describing innovation in broad terms as the implementation of new combinations of service, processes at work, products and markets.

An organization with a strong focus on innovation is characterized by ‘creativity, professional freedom and transformational leadership’ [69]. The findings from this study support this idea. As noted above, IC was found to have the greatest impact on IIB, followed by PsyCap and perceived LAS. Studies in health services research has yet to examine the impact of these three factors collectively. In total, the three factors (LAS, PsyCap and IC) explain 50% of the variance of hospital employees’ IIB, which can be characterized as substantial. Similar to other studies, IC was found to ‘fuel … innovation’ [23] represented by IIB. These findings indicate that if the other two factors (LAS and PsyCap) are present, employees who (cognitively) produce novel and useful ideas are both willing and motivated to (behaviourally) implement them at work.

By including PsyCap and LAS, this study also provides new insight into how personal and organizational factors, individually and collectively, can affect employees’ IC and IIB. To the best of authors’ knowledge, this is one of the few novel studies in health services research to investigate the impact of LAS and PsyCap on IC and IIB. Although both PsyCap and LAS are associated with IC and IIB, there are differences in their impact on the two variables. First, PsyCap shows a significantly greater direct impact on IIB than LAS (β = 0.34 versus β = 0.07). This does not mean that LAS is unimportant for IIB. LAS provides employees with a necessary autonomy and freedom to take the initiative to perform IIB. LAS can thus be characterized as a precondition for IIB. However, autonomy in itself is insufficient to trigger IIB. Employees must also have a personal inner drive to make use of their freedom to perform IIB. The findings from this study indicate that PsyCap is the motivational factor. Consequently, the comparison of the individual impact of LAS and PsyCap highlights that the potential to release employees’ IIB works through their PsyCap. The PsyCap four resources have together a synergistic impact on IIB. This motivational aspect of PsyCap to perform IIB is needed for at least two reasons. First, IIB goes beyond employees’ typical in-role responsibility and accordingly constitutes an extra-role effort. Second, there is always a risk of failure in IIB. Most probably there are also obstacles that one must overcome. However, provided that employees have a satisfactory level of PsyCap. It ‘fuels’ them with energy and goal-directed IIB. The impact of PsyCap on IIB found in this study is supported by previous research [30, 31, 33, 34].

Although LAS has a less direct influence on employees’ IIB than PsyCap, this study found a different pattern in their links to IC. In this situation, LAS and PsyCap have an almost identical impact on IIB (β = 0.26 for LAS and β = 0.32 for PsyCap). IC is a cognitive concept that describes employees’ ‘production of … ideas’ [19]. The findings reveal that LAS significantly promotes employees’ IC. Thinking creatively can be considered a relatively complex task. The literature states that ‘complex tasks … require a higher degree of … autonomy’ [50]. This study supports this statement by empirically illustrating how LAS in healthcare organizations can directly stimulate employees’ IC.

However, PsyCap is also found to be an important driver of IC. This illustrates the multiple roles of PsyCap, which influences both IC and IIB. PsyCap is characterized as openness to change to achieve ‘effective work performance’ [25]. As this study reveals, LAS can influence employees’ PsyCap. Specifically, LAS explains about 30% (*R*^2^ = 0.27) of the variance of PsyCap. Moreover, through the mediation of PsyCap, LAS also simultaneously influences employees’ IC.

Although there are differences in the magnitude of LAS and PsyCap, both are directly linked to IC. On the other hand, the findings reveal how personal factors (IC and PsyCap) and an organizational factor (LAS) functioning in tandem, both directly and indirectly, have a complex symbiotic relationship in promoting IIB. There is a scarcity of studies in health services research that have explored the multifaceted relationship between these factors. The important roles of LAS and PsyCap can be seen through the lens of broaden-and-build theory [70]. Both LAS and PsyCap focus on conditions that stimulate employees’ personal growth, thriving and positive emotions. As this study reveals, when employees view LAS positively, and their level of PsyCap is satisfactory, these two factors work both individually and collectively to increase IC and IIB.

### Implications for practice

The contributions of this study lead to three following practical guidelines for both hospital division managers, as well as hospital unit managers in encouraging positive IIB at work. First, our study encourages healthcare managers, including division managers, department managers and policy maker to view healthcare innovation through a LAS lens. Results of this study shows that LAS seems to have a managing role over employees’ PsyCap, IC and IIB. For healthcare managers, the findings suggest that it is of fundamental importance for healthcare organizations to have co-ordinated and pragmatic leadership. This is expressed well by Hocine and Zhang [47]: ‘Today leaders are more like employee supporters than employee supervisors. Creating intentionally supportive and motivating environment … in the workplace for employees to be creative and innovative is part of modern leadership’ [47]. As such, healthcare managers are encouraged to listen, inspire and motivate their employees, as these fundamental aspects, as shown in the results of this study, improves employees’ PsyCap, IC and IIB.

Second, due to the broad definition used in this study on IIB, innovation can be manifested in all types of hospital work. Consequently, a practical managerial implication for healthcare organizations, as well as hospital managers in various roles, is to not narrow their focus simply to motivating those with a single job (e.g. frontline employees) to perform IIB. In contrast, one should take a broad approach and stimulate all employees’ IIB no matter what their role in the organization. This suggests a need to take a ‘top-down’ perspective on IIB in healthcare organizations. This entails that senior managers of healthcare organizations should try their best to stimulate middle managers’ IIB at work. In the next round, middle managers should do the same for their subordinates, and so forth. This creates a positive and self-reinforcing IIB spiral that could potentially involve the whole organization and lay the foundation of what Mesfin et al. label ‘innovative culture’ [69]. Mesfin et al. found that employees’ perceptions of ‘innovative culture’, regardless of their job, was ‘the most preferred culture type’ [69].

Third, as already mentioned, this study found PsyCap to be a motivational factor. Based on our findings, hospital division managers, as well as hospital unit managers, should be aware of the impact of LAS and navigate employees’ IIB by investing in employees’ PsyCap. Healthcare is a complex system [71], and the findings of this study reveal the many advantages of capitalizing on PsyCap. As such, healthcare managers (both hospital division and hospital unit managers) are encouraged to pay extra attention to employees’ growth and positive emotions at work. For example, healthcare managers are encouraged to show initiative in giving authority in work roles, so that employees are more likely to feel confident in meeting the challenges they face in their work tasks. As a result, employees will be better equipped to contribute creative ideas to solve challenges in their work roles and improve the overall quality of their job.

Additionally, some of the findings in this study are supported by previous studies found in the literature, though previous findings were not reflected in the healthcare environment. This indicates that, although the findings here focused on healthcare organizations, the practical implications for managers independent of healthcare organizations are two-fold. First, managers need to consider the important role of a supportive work environment in their organisation, and the effect this has on employees’ IIB [44]. The supportive work environment of an organization should be monitored and ‘designed’ so that it involves diversity, daily work role challenges in areas such as creativity, skills and knowledge, and where achievable require individuals at work to be involved in problem-solving processes or tasks. Because IIB holds both a cognitive feature as well as behavioural feature [14], managers will benefit from overall human resource development in areas such as creativity, innovativeness, skills and knowledge, inspiring better problem-solving strategies, a sense of ownership among employee’s work roles, but also increasing the overall accountability. Problem-solving tasks should be designed to hold variations in complexity, both for higher levels of innovativeness, but also for overall innovative outcomes. As such, LAS would be a key element in positively affecting employees’ level of IIB at work, because LAS will function as a key driving force. Second, managers are advised to present authoritative opportunities for employees to be challenged [50], but most importantly to improve the interpersonal relationship, with their leaders, within their organization, and across work roles. This is vital for organizations that seek to improve employee’s ability to generate ideas, as well as implement ideas across work roles. In turn, the supportive environment found at a given organization, will function as a promoter for employee’s psychological state of development, and thus ‘spilling over’ innovative outcomes at work*.*

### Limitations and future research

Like most studies, this study has its limitations. However, these limitations offer several opportunities for future research. It is notable that this study makes no distinction between the degree of newness of the innovation, whether the IIB is incremental (e.G. *minor* improvements of service quality) or radical (e.g. the introduction of an entirely new way of providing quality service). Below are seven specific suggestions for future research.

First, as this study looked at a single healthcare organization, its generalizability and robustness in relation to other healthcare organizations are limited. Because the study had a cross-sectional design and used an online survey for data collection, the results might suffer from self-selection bias and inference of causality. As such, future research should employ longitudinal data to examine the potential of the causal relationship among the studied constructs. In addition, future research should explore the variation in leadership roles caused by the complexity of healthcare systems, to assess the differences a leader may experience in a healthcare setting.

Second, leadership is among the most important precursors of innovation. However, more work is needed to identify what leadership style most effectively produces innovation in healthcare organizations. This study limited its focus to a single leadership style as the antecedent to IIB. Although LAS has a significant impact, future research should include other leadership styles. One relatively new leadership style is ambidextrous leadership. Ambidextrous leadership involves two leadership styles, that when interacting with each other, promotes innovation in organizations [72]. There is a scarcity in health services research in examining the impact of ambidextrous leadership on IIB, IC, PsyCap or other factors that potentially associated with innovation. Consequently, more research is needed to reveal the effectiveness of the ambidextrous leadership style and its capability to promote innovative behaviour in healthcare organizations.

Third, as shown in Fig. 1, this study focused on examining the relationship between the studied constructs, LAS, PsyCap, IC and IIB, on a general level. As such, the results are limited to interpretation within the context of common healthcare culture. In addition, this study focused on the personal relationship-dependent IIB, a limitation that prompts a suggestion for future research. Future research may consider a longitudinal research design examining sustainable organizational cultures that promote IIB over time. Future research should also explore the potential loss of stimulus if and when a leader leaves the organization, and may further examine the various levels on the influence of healthcare culture in fostering IIB at work.

Fourth, employees’ PsyCap was found to have a significant impact on both IC and IIB in this study. Consequently, it is important to capitalize on employees’ PsyCap to strengthen an organization’s ability to innovate. This stresses the continuous need to cultivate and manage PsyCap resources. In this study, LAS was found to be one way to do this. However, future research needs to include other factors to explore a broader system construct that addresses positive organizational culture (e.g. an innovative culture or hierarchical culture). Specifically, future research can explore factors such as leadership style (e.g. ambidextrous or authentic leadership), organizational climate (e.g. co-operative or competitive), learning (e.g. team learning or relationship learning), organizational vision integration, organizational commitment and organizational attractiveness.

Fifth, the study did not take into account the complexity of the healthcare setting. In a recent study, Glover et al. examined how the complexity of healthcare influences innovation performance in complex units [71]. The idea of ambidextrous leadership—that “the complexity of innovation activities needs to be matched by an equally complex leadership approach” [72]—provides opportunities for future research in exploring the influence of complex adaptive systems to IIB in healthcare.

Sixth, the study focused on expanding our current understanding of IIB in healthcare organizations. In professional service firms, such as healthcare organizations, empowered employees are found to drive innovation at work while contributing new and novel ideas to face changes and challenges in the current healthcare environment [5, 6]. However, as the focus on IIB in health service research is still in its early stages, there are great opportunities for future studies. For instance, this study did not explore in detail how IIB, when implemented, can be a strength for work processes or complex work systems. Future research can therefore qualitatively explore IIB in healthcare organizations, to determine its specific justifications and provide examples of the value of IC and IIB when implemented in work processes and work systems, locally or in the overall organization.

Seventh and lastly, the concept of ‘thriving’ [73] has recently been proposed as a promising and important aspect for organizations. Thriving at work is defined as a ‘psychological state in which individuals experience both a sense of vitality and learning at work’ [74]. Studies in health services research have yet to explore the connection of employees’ perceptions of thriving to PsyCap. Interestingly, thriving has also been directly linked to IIB. Riaz et al. found this strong linkage between employees’ thriving at work and their innovative work behaviour [75]. However, scarce studies have examined these relationships in an healthcare context. This indicates great potential and opportunities for future research.

## Conclusions

This study contributes to our understanding of innovation in healthcare organizations from the perspective of individual employees. Specifically, it reveals a multifaceted association between IIB, LAS, PsyCap and IC. From a leadership perspective, the findings highlight the core role of LAS in promoting employees’ innovative behaviour in healthcare organizations.

## Supplementary Information

**Additional file 1.** Appendix 1. Questionnaire developed for this study.

## Data Availability

The datasets used and/or analysed during the current study are available from the corresponding author on reasonable request.

## Abbreviations

IIB: Individual innovative behaviour
IC: Individual creativity
LAS: Leadership autonomy support
PsyCap: Psychological capital
STD: Self-determination theory
SEM: Structural equation modelling
AVE: Average variance extracted
NSD: Norwegian Social Science Data Services
DPO: Data Protection Official
